# Mixed-Sequence Recognition of Double-Stranded DNA Using Enzymatically Stable Phosphorothioate Invader Probes

**DOI:** 10.3390/molecules200813780

**Published:** 2015-07-29

**Authors:** Brooke A. Anderson, Saswata Karmakar, Patrick J. Hrdlicka

**Affiliations:** Department of Chemistry, University of Idaho, Moscow, ID 83844-2343, USA; E-Mails: brookea@scripps.edu (B.A.A.); saswatak@stanford.edu (S.K.)

**Keywords:** DNA recognition, oligonucleotides, pyrene

## Abstract

Development of probes that allow for sequence-unrestricted recognition of double-stranded DNA (dsDNA) continues to attract much attention due to the prospect for molecular tools that enable detection, regulation, and manipulation of genes. We have recently introduced so-called Invader probes as alternatives to more established approaches such as triplex-forming oligonucleotides, peptide nucleic acids and polyamides. These short DNA duplexes are activated for dsDNA recognition by installment of +1 interstrand zippers of intercalator-functionalized nucleotides such as 2′-*N*-(pyren-1-yl)methyl-2′-*N*-methyl-2′-aminouridine and 2′-*O*-(pyren-1-yl)methyluridine, which results in violation of the nearest neighbor exclusion principle and duplex destabilization. The individual probes strands have high affinity toward complementary DNA strands, which generates the driving force for recognition of mixed-sequence dsDNA regions. In the present article, we characterize Invader probes that are based on phosphorothioate backbones (PS-DNA Invaders). The change from the regular phosphodiester backbone furnishes Invader probes that are much more stable to nucleolytic degradation, while displaying acceptable dsDNA-recognition efficiency. PS-DNA Invader probes therefore present themselves as interesting probes for dsDNA-targeting applications in cellular environments and living organisms.

## 1. Introduction

The promise of molecular tools that enable detection, regulation, and manipulation of genes, continues to inspire nucleic acid chemists to develop probes for sequence-unrestricted recognition of double-stranded DNA (dsDNA) [1,2,3,4,5]. Pioneering approaches include triplex-forming oligonucleotides (TFOs) [1,6], peptide nucleic acids (PNAs) [3,7], and minor-groove binding polyamides [5,8,9]. However, these probes display limitations, which has prevented their wide-spread adoption. Thus, TFOs and standard PNAs only bind to polypurine sites, which limits the number of available target regions. Moreover, most PNA-based approaches require non-physiologic salinity for efficient dsDNA recognition to take place, while polyamides typically only recognize short target sequences, which renders identification of unique target regions challenging. Several alternative strategies for dsDNA recognition have therefore been developed including TFOs with engineered nucleobases [10,11], pseudocomplementary DNA (pcDNA) [12], pcPNA [4,13,14], and γ-PNAs [15,16]. Whilst these probes have more relaxed sequence requirements, they exhibit other limitations. More recently, approaches based on artificial nucleases have gained tremendous attention [2,17]. However, it is clear that many future dsDNA targeting applications will benefit from more simple and inexpensive hybridization-based probes that enable rapid, potent, and specific recognition of mixed-sequence dsDNA under physiologic conditions.

**Figure 1:**
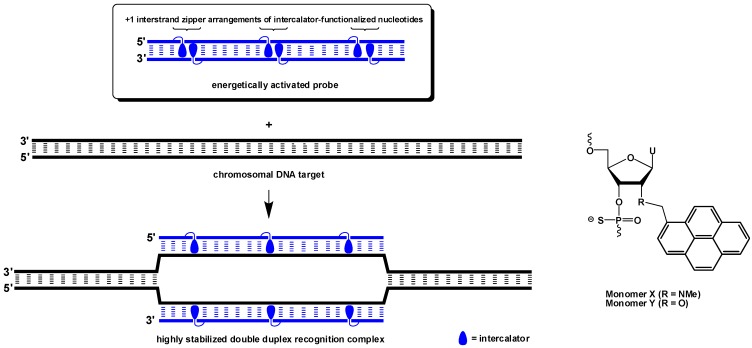
Illustration of the Invader approach for recognition of mixed-sequence dsDNA and structures of monomers used herein. Droplets denote intercalating pyrene moieties.

We have recently presented an alternative approach, which is based on short DNA duplexes that are energetically activated for mixed-sequence recognition of dsDNA through installment of +1 interstrand zippers of intercalator-functionalized nucleotides such as 2′-*N*-(pyren-1-yl)methyl-2′-*N*-methyl-2′-aminouridine monomer **X** or 2′-*O*-(pyren-1-yl)methyluridine monomer **Y** (Figure 1—for a definition of the zipper nomenclature, see the Experimental Section) [18,19]. This structural motif forces the intercalating pyrene moieties into the same region of the duplex [20,21], leading to violation of the “nearest neighbor exclusion principle” [22,23] and destabilization of the duplex. According to the principle, intercalators bind with a maximum loading of one ligand per two base-pairs due to limitations in local helix expandability (every intercalation event unwinds the duplex by ~3.4 Å) [24] and/or to avoid disruption of highly stable stacking interactions between nucleobases and the first bound intercalator [25]. Conversely, the two probe strands have very high affinity toward complementary DNA as duplex formation results in strongly stabilizing π-stacking interactions between the intercalator and neighboring base pairs. The difference in thermodynamic stability between the probe duplexes and recognition complexes provides the driving force that allows for recognition of mixed-sequence chromosomal DNA targets [18,19]. Our previous efforts to optimize the dsDNA-recognition efficiency of these so-called Invader probes have focused on varying: the number and relative position of the key activating monomers, the nature of the nucleobase and intercalator, and the length of the linker and the orientation between the intercalator and sugar skeleton [19,20,21,26,27,28].

In the present study, we set out to study **X**- or **Y**-modified Invader probes that are based on a phosphorothioate backbone (PS-DNA Invaders). Phosphorothioate linkages are well-known to increase the nucleolytic stability of oligonucleotides (ONs) [29], which has enabled cell culture and *in vivo* studies [30]. However, phosphorothioate linkages are also known to decrease the affinity of oligonucleotides towards their complementary DNA and RNA targets (cDNA/cRNA) [31]. Given that the use of phopsphorothioate-based Invader probes likely will result in even more labile probes (presumably favorable), but also less thermodynamically stable recognition complexes (presumably unfavorable), it is not immediately clear how the dsDNA-recognition efficiency of PS-DNA Invaders will compare to conventional Invader probes that are based on phosphodiester backbones. We therefore synthesized a series of PS-DNA Invader probes, which were characterized by means of thermal denaturation experiments, UV-vis absorption and fluorescence spectroscopy, and dsDNA recognition and enzymatic stability experiments. The insight gained from this study is valuable for future cell culture experiments involving Invader probes.

## 2. Results and Discussion

### 2.1. Synthesis of Modified ONs and Experimental Design

The corresponding phosphoramidites of monomers **X** and **Y** were synthesized as previously described [27,32] and used to prepare modified PS-DNA strands via machine-assisted DNA synthesis and the following hand-coupling conditions (coupling time; activator; coupling yield): **X** (15 min; 5-[3,5-bis(trifluoromethyl)phenyl]-1*H*-tetrazole; ~99%) and **Y** (15 min; 4,5-dicyanoimidazole; ~99%). Sulfurization was carried out using 3-((*N*,*N*-dimethylaminomethylidene)amino)-3*H*-1,2,4-dithiazole-5-thione. The identity and purity of all strands was established via MALDI-TOF (Table S1) and ion-pair reverse phase HPLC (>80% purity), respectively.

In order to delineate the role of backbone chemistry, **X**- and **Y**-modified PS-DNA were studied in the same 9- and 13-mer nucleotide sequence contexts that were used to study the corresponding phosphodiester DNA (PO-DNA) probes [19,32]. PS-DNA with a single incorporation of monomer **X** or **Y** in the 5′-G**B**G ATA TGC context are denoted **X1** and **Y1**, respectively. Similar conventions apply for the **B2**–**B6** series of 9-mer PS-DNA and the **B7**–**B14** series of 13-mer PS-DNA (Table 1).

**Table 1:** Change in thermal denaturation temperature (Δ*T*_m_) of duplexes between **X-**/**Y**-modified PS-DNA **B1**–**B14** and cDNA/cRNA, relative to duplexes between reference PS-DNA and cDNA/cRNA ^a^.

ON	PS-DNA Sequence	B =	Δ*T*_m_ (°C)			
			+cDNA		+cRNA	
			X	Y	X	Y
**B1**	5′-G**B**G ATA TGC		+5.0	+4.5	−4.0	−4.0
**B2**	5′-GTG A**B**A TGC		+13.0	+12.0	±0.0	±0.0
**B3**	5′-GTG ATA **B**GC		+8.0	+5.5	−4.0	−4.0
**B4**	3′-CAC **B**AT ACG		+4.0	+2.0	−5.0	−5.5
**B5**	3′-CAC TA**B** ACG		+12.5	+11.0	+3.0	+1.5
**B6**	3′-CAC **B**A**B** ACG		+11.0	+10.5	<−8.0	−6.5
**B7**	5′-GG**B** ATA TAT AGG C		+6.0	+5.5	-	-
**B8**	3′-CCA **B**AT ATA TCC G		+9.5	+9.0	-	-
**B9**	5′-GG**B** A**B**A TAT AGG C		+12.0	+12.5	-	-
**B10**	3′-CCA **B**A **B**ATA TCC G		+16.0	+16.0	-	-
**B11**	5′- GGT A**B**A **B**AT AGG C		+16.5	+16.0	-	-
**B12**	3′- CCA TA**B** A**B**A TCC G		+18.0	+17.5	-	-
**B13**	5′-GG**B** ATA TA**B** AGG C		+15.5	+13.5	-	-
**B14**	3′-CCA **B**AT ATA **B**CC G		+18.0	+16.0	-	-

^a^ **B1**–**B3** are measured relative to 5′-[PS-DNA]-GTGATATGC (*T*_m_ = 19.5 °C and 18.0 °C with cDNA and cRNA, respectively); **B4**–**B6** are measured relative to 3′-[PS-DNA]-CACTATACG (*T*_m_ = 19.5 °C and 18.0 °C with cDNA and cRNA, respectively); **B7**/**B9**/**B11**/**B13** are measured relative to 5′-[PS-DNA]-GGTATATATAGGC (*T*_m_ = 29.0 °C with cDNA) and **B8**/**B10**/**B12**/**B14** are measured relative to 3′-[PS-DNA]-CCATATATATCCG (*T*_m_ = 26.5 °C with cDNA). *T*_m_’s are determined as the maximum of the first derivative of melting curves (*A*_260_ *vs.* *T*) recorded in medium salt phosphate buffer ([Na^+^] = 110 mM, [Cl^−^] = 100 mM, pH 7.0 (NaH_2_PO_4_/Na_2_HPO_4_)), using 1.0 µM of each strand. Reported *T*_m_’s are averages of at least two measurements within 1.0 °C; A = adenin-9-yl PS-DNA monomer, C = cytosin-1-yl PS-DNA monomer, G = guanin-9-yl PS-DNA monomer, T = thymin-1-yl PS-DNA monomer. For structures of monomers **X** and **Y**, see Figure 1. “-” denotes not determined.

### 2.2. Thermal Denaturation Properties of **X**-/**Y**-Modified PS-DNA

Thermal denaturation temperatures (*T*_m_’s) of duplexes between **X**- or **Y**-modified PS-DNA and cDNA/cRNA were determined in medium salt phosphate buffer ([Na^+^] = 110 mM) and compared to duplexes between unmodified PS-DNA and cDNA/cRNA. The resulting denaturation curves display monophasic sigmoidal transitions as expected (Figure S1). Introduction of **X** or **Y** monomers into PS-DNA strands results in the formation of very stable duplexes with cDNA (Δ*T*_m_/modification between +2.0 and +13.0 °C, Table 1), while duplexes with cRNA are far less stable (Δ*T*_m_/modification between <−8.0 to +3.0 °C, Table 1). Monomer **X** induces slightly higher cDNA affinity than monomer **Y**, and PS-DNA strands in which the pyrene-functionalized monomer is flanked by a 3′-purine result in greater duplex stabilization than strands with 3′-flanking pyrimidines (e.g., compare Δ*T*_m_’s for **B1** and **B4**, Table 1). The sequence dependence, along with the prominent DNA selectivity (Table S2), are characteristic properties of oligonucleotides modified with intercalating pyrene moieties [20,21,33], and mirror our observations with **X**-/**Y**-modified PO-DNA strands [19,27,32], except that the stabilizing effects of monomers **X** and **Y** are slightly lower when introduced in PS-DNA strands (*i.e.*, Δ*T*_m_/modification are 0–3 °C lower). As expected, [31] the absolute *T*_m_’s of duplexes between **X**-/**Y**-modified PS-DNA and cDNA are ~1 °C lower per phosphorothioate linkage relative to the corresponding duplexes with **X**-/**Y**-modified PO-DNA [19,32,34].

### 2.3. Photophysical Properties of X-/Y-Modified PS-DNA

**Figure 2:**
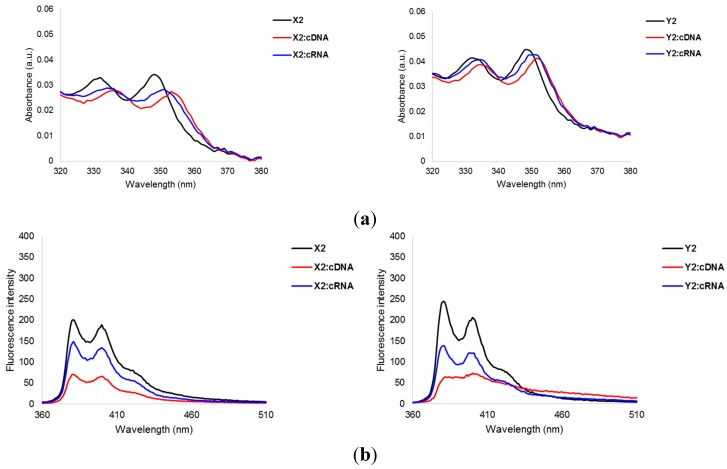
(**a**) Absorbance spectra of representative PS-DNA strands **X2** and **Y2** and the corresponding duplexes with cDNA/cRNA. Spectra were recorded at *T* = 5 °C; (**b**) Steady-state fluorescence emission spectra of **X2** and **Y2** and the corresponding duplexes with cDNA/cRNA. Spectra were recorded at 10 °C using λ_ex_ = 350 nm. Each strand was used at 1.0 μM concentration in *T*_m_ buffer. For additional spectra, see Figures S2 and S3.

UV-Vis absorption and steady-state fluorescence emission spectra of 9-mer **X**- or **Y**-modified ONs were recorded in the absence or presence of cDNA/cRNA to verify the intercalative binding mode of the pyrene moieties. Indeed, hybridization with cDNA is accompanied by prominent hypochromic and bathochromic shifts in pyrene absorption maxima (Δλ_max_ = 0–5 nm, Figure 2, Figures S2 and S3; Table S5), which is indicative of ground-state electronic interactions between pyrenes and nucleobases [35] and intercalation. Less prominent bathochromic shifts are observed upon hybridization with cRNA, most likely since intercalation into more compressed RNA-like duplexes is less favorable [20,21,33]. More pronounced bathochromic shifts are generally observed with **X**-modified PS-DNA strands, a trend that also was seen with the corresponding PO-DNA probes [32]. We speculate that this is due to particularly efficient overlap between the pyrene moiety and the neighboring base pairs but the underlying reasons could also be of an electronic nature.

Steady-state fluorescence emission spectra (λ_ex_ = 350 nm, *T* = 10 °C) of these duplexes display two vibronic bands at λ_em_ = 381 ± 1 nm and 400 ± 1 nm, respectively, in addition to a small shoulder at ~420 nm (Figure 2 and Figure S4). Hybridization with cDNA/cRNA is accompanied by 1.4- to 4.6-fold decreases in fluorescence intensity, with greater decreases being observed upon cDNA binding. Hybridization-induced decreases in pyrene emission are often observed with probes where intercalation, and quenching of fluorescence by neighboring nucleobases, is likely [36,37,38].

The photophysical trends strongly suggest that the pyrene moieties of the **X** and **Y** monomers maintain their intercalative binding modes when incorporated in PS-DNA strands, which is a prerequisite for their use as structural elements of Invader probes.

### 2.4. Thermal Denaturation Properties of PS-DNA Duplexes with Interstrand Zipper Arrangements of **X** or **Y** Monomers

Next, the *T*_m_’s of PS-DNA duplexes with different interstrand zipper arrangements of monomers **X** and **Y** was determined (Table 2). Consistent with our observations in the corresponding PO-DNA series [20], PS-DNA duplexes with +4 or −1 interstrand zipper arrangements of **X** or **Y** monomers are very stable. In contrast, 9-mer PS-DNA duplexes with +2 and +1 interstrand zippers are very labile (no observable transitions above 10 °C), presumably because the nearest neighbor exclusion principle is violated. 13-mer PS-DNA duplexes with one +1 interstrand zipper of **X** or **Y** monomers or two sequential +1 interstrand zippers of **X** monomers are also labile (see Δ*T*_m_ for **X7**:**X8**, **X9**:**X10**, **X11**:**X12** and **Y7**:**Y8**, Table 2), whereas PS-DNA duplexes with two +1 interstrand zipper arrangements of **Y** monomers are moderately stabilized, in a similar manner as in the corresponding PO-DNA series [19]. As expected, the 13-mer PS-DNA Invader probes display much lower absolute *T*_m_’s than the corresponding PO-DNA based Invader probes (PS-DNA *T*_m_ = 12.5–26.0 °C, Table 2 *vs.* PO-DNA *T*_m_ = 33.5–45.0 °C, reference [19]), which is expected to facilitate probe dissociation.

**Table 2:** *T*_m_ and Δ*T*_m_ values for PS-DNA duplexes with different interstrand zipper arrangements of **X** and **Y** monomers. ^a^

ON	ZP	PS-DNA Duplex	B =	*T*_m_ (Δ*T*_m_)/°C	
				X	Y
**B1**	+4	5′-G**B**G ATA TGC		29.5	29.5
**B5**		3′-CAC TA**B** ACG		(>+19.5)	(>+19.5)
**B1**	+2	5′-G**B**G ATA TGC		<10.0	<10.0
**B4**		3′-CAC **B**AT ACG			
**B2**	+1	5′-GTG A**B**A TGC		<10.0	<10.0
**B5**		3′-CAC TA**B** ACG			
**B2**	−1	5′-GTG A**B**A TGC		22.0	19.0
**B4**		3′-CAC **B**AT ACG		(>+12.0)	(>+9.0)
**B7**	+1	5′-GG**B** ATA TAT AGG C		13.5	12.5
**B8**		3′-CCA **B**AT ATA TCC G		(−4.0)	(−5.0)
**B9**	+1	5′-GG**B** A**B**A TAT AGG C		17.5	23.5
**B10**		3′-CCA **B**A **B**ATA TCC G		(±0.0)	(+6.0)
**B11**	+1	5′- GGT A**B**A **B**AT AGG C		18.0	26.0
**B12**		3′- CCA TA**B** A**B**A TCC G		(+0.5)	(+8.5)
**B13**	+1	5′-GG**B** ATA TA**B** AGG C		24.5	24.0
**B14**		3′-CCA **B**AT ATA **B**CC G		(+7.0)	(+6.5)

^a^ Δ*T*_m_ = change in *T*_m_ relative to reference duplexes 5′-[PS-DNA]-GTGATATGC:3′-[PS-DNA]-CACTATACG (*T*_m_ < 10.0 °C) and 5′-[PS-DNA]-GGTATATATAGGC:3′-[PS-DNA]-CCATATATATCCG (*T*_m_ = 17.5 °C). See Table 1 for experimental conditions. ZP = zipper.

### 2.5. Recognition of DNA Hairpins Using Energetically Activated Probe Duplexes

**Figure 3:**
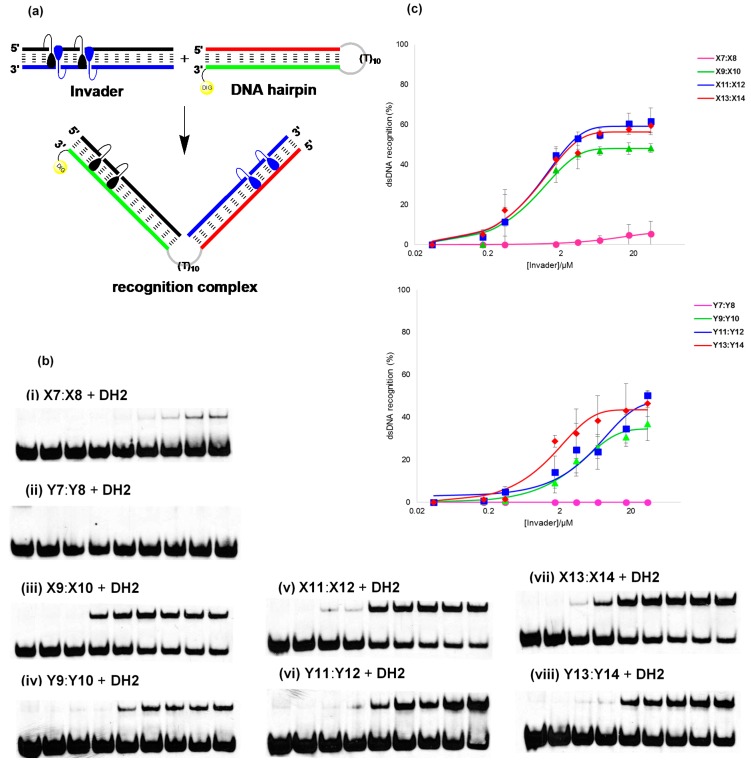
Recognition of dsDNA model target **DH2** using different Invader probes. (**a**) Illustration of recognition process; (**b**) representative electrophoretograms for recognition of **DH2** using no (lane 1) or 1-/5-/10-/50-/100-/200-/500-/1000-fold excess (lanes 2–9) of **X** and **Y-**modified 13-mer Invader probes (i–viii); (**c**) dose-response curves (average of at least three independent experiments, error bars represent standard deviation). Experimental conditions for electrophoretic mobility shift assay: separately pre-annealed targets (34.4 nM) and probes (variable concentrations) were incubated for 12–16 h at room temperature in 1X HEPES buffer (50 mM HEPES, 100 mM NaCl, 5 mM MgCl_2_, 10% sucrose, 1.4 mM spermine tetrahydrochloride, pH 7.2) and then run on 16% non-denaturing PAGE (70 V, 2.5 h, ~4 °C) using 0.5× TBE as a running buffer (45 mM Tris, 45 mM boric acid, 1 mM EDTA); DIG: digoxigenin.

The energetically activated nature of the Invader probes, coupled with the high cDNA affinity of the individual probe strands, suggests that recognition of isosequential dsDNA target regions should be thermodynamically favorable (see also Table S6). To study this in greater detail, we targeted 9- and 13-mer PS-DNA Invader probes against **DH1** and **DH2**, *i.e.*, two different 3′-digoxigenin (DIG) labeled DNA hairpins (DH), which are comprised of 9-mer or 13-mer double-stranded target regions that are connected by a T_10_ loop (Figure 3 and Figure S5). Room-temperature incubation of **DH1** with 9-mer PS-DNA Invader probes **X2**:**X5** or **Y2**:**Y5** did not result in formation of recognition complexes as evidenced by the absence of slower migrating bands on non-denaturing PAGE gels, even when using 500-fold probe excess (Figure S5b). Annealing of **DH1** with excess **X2**:**X5** or **Y2**:**Y5** followed by room temperature incubation (*i.e.*, forcing conditions), also did not result in formation of recognition complexes (Figure S5c) presumably as they are not sufficiently stable at the experimental conditions (note, *T*_m_’s for duplexes between **X2**/**X5**/**Y2**/**Y5** and cDNA are only 30.5–32.5 °C, Table 1).

Room temperature incubation of **DH2** with 13-mer PS-DNA Invader probes containing one +1 interstrand zipper arrangement of **X** or **Y** monomers resulted in no or very little dsDNA recognition (Figure 3b). However, dose-dependent formation of a slower migrating band is observed when PS-DNA Invader probes containing two energetic hotspots are used (Figure 3b). Thus, between 24%–56% dsDNA-recognition is observed when these probes are used at 200-fold molar excess. **X**-modified PS-DNA probes recognize **DH2** more efficiently than **Y**-modified probes (46%–56% *vs*. 24%–38%, respectively, Figure 3c), which is consistent with the former being energetically more strongly activated for dsDNA recognition (*i.e.*, larger stability differences between probe-target duplexes and probe duplexes, see Table S6). **X11**:**X12** and **X13**:**X14** are the most efficient probes in this series, with *C*_50_ values of ~2.6 μM and ~2.9 μM, respectively (*C*_50_ denotes the probe concentration resulting in 50% recognition).

The 13-mer PS-DNA Invader probes generally recognize **DH2** less efficiently than the corresponding PO-DNA Invader probes (Figure S6). Considering that: (i) PS-DNA and PO-DNA based Invader probes are similarly activated for dsDNA-recognition (Table S6); (ii) PS-DNA Invader probes display markedly lower *T*_m_’s than PO-DNA Invader probes; and (iii) duplexes between individual PS-DNA Invader probe strands and cDNA display significantly lower *T*_m_’s than duplexes between PO-DNA Invader probe strands and cDNA, it appears that high cDNA affinity of the individual probe strands is a more important factor for efficient dsDNA-recognition than low *T*_m_’s of Invader probes.

### 2.6. Enzymatic Stability of Individual Invader Strands

**Figure 4:**
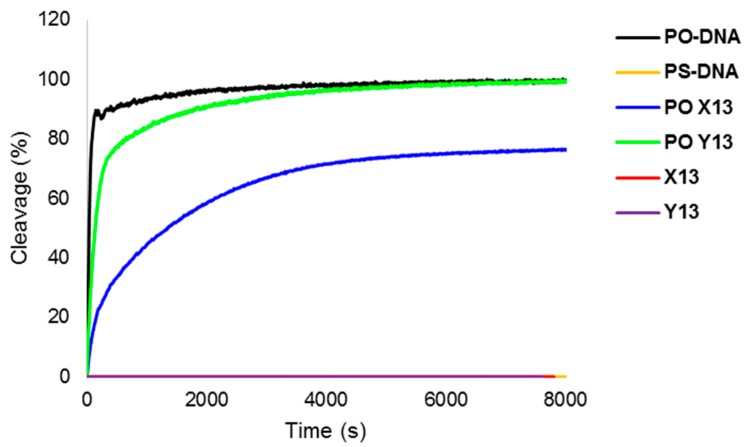
3′-Exonuclease degradation of individual Invader and reference strands. Experiments were performed at 37 °C in magnesium buffer (200 μL solution, 50 mM Tris-HCl, 10 mM Mg^2+^, pH 9.0) using [ON] = 3.3 μM and 0.03 U of snake venom phosphodiesterase. Note, yellow, red and purple lines are superimposed on the X-axis.

Lastly, we studied the stability of individual Invader strands against snake venom phosphordiesterase (SVPDE—a 3′-exonuclease), as a significant proportion of PS-DNA Invader probes are likely to be dissociated at biologically relevant experimental temperatures. Gratifyingly, **X**- and **Y**-modified PS-DNA **X13** and **Y13** are completely stable against SVPDE (Figure 4). In notable contrast, the PO-DNA analog of **Y13** is only degraded slightly more slowly than the corresponding unmodified PO-DNA (95% degradation after ~50 min and ~15 min, respectively, Figure 4), while the PO-DNA analog of **X13** is moderately stable (~95% degradation after ~21 h). The latter observation suggests that the 2′-*N*-methyl-2′-amino-DNA skeleton of monomer **X** confers better protection against 3′-exonucleases than the RNA skeleton of monomer **Y**, which renders them as particularly interesting building blocks for Invader-based DNA-targeting applications in cellular environments [39].

## 3. Experimental Section

### 3.1. Protocol—Synthesis and Purification of ONs

The modified PS-DNA strands were synthesized on a 0.2 μmol scale using a DNA synthesizer, succinyl linked LCAA-CPG (long chain alkyl amine controlled pore glass) columns with a pore size of 500 Å, and standard protocols for incorporation of A^Bz^, C^Bz^, G^iBu^ and T DNA phosphoramidites. The following hand-coupling conditions were used for incorporation of monomers **X** and **Y** (coupling time; activator; coupling yield): **X** (15 min; 5-[3,5-bis(trifluoromethyl)phenyl]-1*H*-tetrazole; ~99%) and **Y** (15 min; 4,5-dicyanoimidazole; ~99%). Modified phosphoramidites were used at 50-fold molar excess and 0.05 M concentration in anhydrous CH_3_CN. Sulfurization was carried out using 3-((*N*,*N*-dimethylaminomethylidene)amino)-3*H*-1,2,4-dithiazole-5-thione (Sulfurizing Reagent II, Glen Research, Sterling, VA, USA) following manufacturer’s specifications (~2 min). Cleavage from solid support and removal of protecting groups was accomplished using 32% aq. ammonia (55 °C, 16–24 h). ONs were purified in the DMT-on mode via ion-pair reverse phase HPLC (C18 column) using a 0.05 M triethylammonium acetate—water/acetonitrile gradient. This was followed by detritylation (80% aq. AcOH) and precipitation (NaOAc/NaClO_4_/acetone, −18 °C for 12–16 h). The identity of synthesized ONs was established through MALDI-MS analysis (Table S1) recorded in positive ions mode on a quadrupole time-of-flight tandem mass spectrometer equipped with a MALDI source using anthranilic acid, 3-hydroxypicolinic acid (3-HPA) or 2′,4′,6′-trihydroxyacetophenone (THAP) as matricies. Purity was verified by ion-pair reverse phase HPLC running in analytical mode (>80%).

### 3.2. Protocol—Thermal Denaturation Studies

ON concentrations were estimated using the following extinction coefficients for DNA (OD/μmol): G (12.01), A (15.20), T (8.40), C (7.05); RNA (OD/μmol): G (13.70), A (15.40), U (10.00), C (9.00); pyrene (22.4) [40]. Strands were thoroughly mixed and denatured by heating to 70–85 °C, followed by cooling to the starting temperature of the experiment. Quartz optical cells with a path length of 1.0 cm were used. *T*_m_’s of duplexes (1.0 µM final concentration of each strand) were measured using a UV/Vis spectrophotometer equipped with a Peltier temperature controller and determined as the maximum of the first derivative of thermal denaturation curves (*A*_260_ *vs.* *T*) recorded in medium salt phosphate buffer (*T*_m_ buffer: 100 mM NaCl, 0.1 mM EDTA and pH 7.0 adjusted with 10 mM Na_2_HPO_4_ and 5 mM Na_2_HPO_4_). The temperature of the denaturation experiments ranged from at least 15 °C below *T*_m_ to 20 °C above *T*_m_ (although not below 2 °C). A temperature ramp of 0.5 °C/min was used in all experiments. Reported *T*_m_’s are averages of two experiments within ±1.0 °C.

### 3.3. Protocol—Absorption Spectra

UV-vis absorption spectra (range 200–600 nm) were recorded at 5 °C using the same samples and instrumentation as in the thermal denaturation experiments.

### 3.4. Protocol—Steady-State Fluorescence Emission Spectra

Steady-state fluorescence emission spectra of **X**- or **Y**-modified ONs and the corresponding duplexes with complementary DNA/RNA targets, were recorded in non-deoxygenated thermal denaturation buffer (each strand at 1.0 μM concentration) and obtained as an average of five scans using an excitation wavelength of λ_ex_ = 350 nm. Excitation and emission slits of 5.0 nm and 2.5 nm, respectively, were used along with a scan speed of 600 nm/min. Experiments were conducted at 10 °C.

### 3.5. Protocol—Electrophoretic Mobility Shift Assay

This assay was performed as previously described [18]. Unmodified DNA hairpins **DH1** and **DH2** were obtained from commercial sources and used without further purification. The DNA hairpins were 3′-DIG-labeled using the 2^nd^ generation DIG Gel Shift Kit (Roche Applied Bioscience, Madison, WI, USA) following the manufacturer’s recommendation. DIG-labeled ONs obtained in this manner were diluted and used without further purification in the recognition experiments. Pre-annealed probes (85 °C for 10 min, cooled to room temperature over 15 min) and DIG-labeled DNA hairpins (34.4 nM) were mixed and incubated in HEPES buffer (50 mM HEPES, 100 mM NaCl, 5 mM MgCl_2_, 10% sucrose, 1.44 mM spermine tetrahydrochloride, pH 7.2) overnight (12–16 h) at ambient temperature. The reaction mixtures were then diluted with 6× DNA loading dye (Fermentas, Waltham, MA, USA) and loaded onto a 16% non-denaturing polyacrylamide gel. Electrophoresis was performed using a constant voltage of 70 V for 2.5 h at ~4 °C using 0.5× TBE as a running buffer (45 mM Tris, 45 mM boric acid, 1 mM EDTA). Gels were blotted onto positively charged nylon membranes (Roche Applied Bioscience) using constant voltage with external cooling (100 V, ~4 °C). The membranes were exposed to anti-digoxigenin-AP F_ab_ fragments as recommended by the manufacturer of the DIG Gel Shift Kit, transferred to a hybridization jacket, and incubated with the substrate (CSPD) in detection buffer for 10 min at 37 °C. The chemiluminescence of the formed product was captured on X-ray film, which was developed using an X-Omatic 1000A X-ray film developer (Kodak, Rochester, NY, USA). The resulting bands were quantified using Image J software. Invasion efficiency was determined as the intensity ratio between the recognition complex band and the total lane. An average of three independent experiments is reported along with standard deviations. Non-linear regression was used to fit data points from dose-response experiments, using a script written for the “Solver” module in Microsoft Office Excel [41].

### 3.6. Protocol—3′-Exonuclease Stability Assay

The stability of ONs against SVPDE (snake venom phosphodiesterase, Worthington Biochemical Corporation, Lakewood, NJ, USA) was determined by monitoring the increase in absorbance at 260 nm as a function of time. SVPDE dissolved in H_2_O (1.27 μL, 0.52 μg, 0.03 U) was added to a solution of ON (3.3 μM) in magnesium buffer (200 μL, 50 mM Tris·HCl, 10 mM MgCl_2_, pH 9.0) at 37 °C.

### 3.7. Definition of Zipper Nomenclature

The following nomenclature describes the relative arrangement between two pyrene-functionalized monomers positioned on opposing strands in a duplex. The number *n* describes the distance measured in number of base pairs and has a positive value if a monomer is shifted toward the 5′-side of its own strand relative to a second reference monomer on the other strand. Conversely, *n* has a negative value if a monomer is shifted toward the 3′-side of its own strand relative to a second reference monomer on the other strand.

## 4. Conclusions

Invader probes, which are based on phosphorothioate DNA backbones and +1 interstrand zipper arrangements of 2′-*N*-(pyren-1-yl)methyl-2′-*N*-methyl-2′-aminouridine or 2′-*O*-(pyren-1-yl)methyluridine monomers, exhibit high enzymatic stability and affinity toward mixed-sequence dsDNA targets. These characteristics render phosphorothioate-based Invader probes as interesting entities for mixed-sequence DNA recognition applications in cellular environments.

## Supplementary Materials

The supplementary material contains the following: Table S1: MALDI-MS of modified ONs; Figure S1: Representative thermal denaturation curves; Table S2: DNA selectivity of **X**- and **Y**-modified 9-mer ONs; Table S3: Discrimination of mismatched DNA targets by **X2** and **Y2**; Table S4: Discrimination of mismatched DNA targets by **X6** and **Y6**; Figure S2: Absorption spectra of **X1**-**X6** in absence or presence of cRNA or cRNA; Figure S3: Absorption spectra of **Y1**–**Y6** in absence or presence of cRNA or cRNA; Table S5: Absorbance of **X** and **Y** 9-mers in absence or presence of cDNA or cRNA; Figure S4: Steady-state fluorescence emission spectra of **X5** and **Y5** in absence or presence of cDNA or cRNA; Table S6: Thermal advantage (*TA*) values for 13-mer PS-DNA/PO-DNA Invader probes; Figure S5: PS 9-mer invader mediated recognition of DNA hairpin assay; Figure S6: Invader mediated recognition of DNA hairpins of representative PO **X** and **Y**-modified 13-mer invaders; DNase I protocol and discussion; Figure S7: Nuclease susceptibility of PO-Invaders in the presence of DNase I.

Supplementary materials can be accessed at: http://www.mdpi.com/1420-3049/20/08/13780/s1.

## References

[1] Rogers F.A., Lloyd J.A., Glazer P.M. (2005). Triplex-forming oligonucleotides as potential tools for modulation of gene expression. Curr. Med. Chem. Anti- Cancer Agents.

[2] Ghosh I., Stains C.I., Ooi A.T., Segal D.J. (2006). Direct detection of double-stranded DNA: Molecular methods and applications for DNA diagnostics. Mol. Biol. Syst..

[3] Nielsen P.E. (2010). Peptide Nucleic Acids (PNA) in chemical biology and drug discovery. Chem. Biodivers..

[4] Aiba Y., Sumaoka J., Komiyama M. (2011). Artificial DNA cutters for DNA manipulation and genome engineering. Chem. Soc. Rev..

[5] Vaijayanthi T., Bando T., Pandian G.N., Sugiyama H. (2012). Progress and prospects of pyrrole-imidazole polyamide-fluorophore conjugates as sequence-selective DNA probes. Chem. Biol. Chem..

[6] Duca M., Vekhoff P., Oussedik K., Halby L., Arimondo P.B. (2008). The triple helix: 50 Years later, the outcome. Nucleic Acids Res..

[7] Nielsen P.E., Egholm M., Berg R.H., Buchardt O. (1991). Sequence-selective recognition of DNA by strand displacement with a thymine-substituted polyamide. Science.

[8] Dervan P.B., Edelson B.S. (2003). Recognition of the DNA minor groove by pyrrole-imidazole polyamides. Curr. Opin. Struct. Biol..

[9] Blackledge M.S., Melander C. (2013). Programmable DNA-binding small molecules. Bioorg. Med. Chem..

[10] Rusling D.A., Powers V.E.C., Ranasinghe R.T., Wang Y., Osborne S.D., Brown T., Fox K. (2005). Four base recognition by triplex-forming oligonucleotides at physiological pH. Nucleic Acids Res..

[11] Hari Y., Obika S., Imanishi T. (2012). Towards the sequence-selective recognition of double-stranded DNA containing pyrimidine-purine interruptions by triplex-forming oligonucleotides. Eur. J. Org. Chem..

[12] Kutyavin I.V., Rhinehart R.L., Lukhtanov E.A., Gorn V.V., Meyer R.B., Gamper H.B. (1996). Oligonucleotides containing 2-aminoadenine and 2-thiothymine act as selectively binding complementary agents. Biochemistry.

[13] Lohse J., Dahl O., Nielsen P.E. (1999). Double duplex invasion by peptide nucleic acid: A general principle for sequence-specific targeting of double-stranded DNA. Proc. Natl. Acad. Sci. USA.

[14] Ishizuka T., Yoshida J., Yamamoto Y., Sumaoka J., Tedeschi T., Corradini R., Sforza S., Komiyama M. (2008). Chiral introduction of positive charges to PNA for double-duplex invasion to versatile sequences. Nucleic Acids Res..

[15] Rapireddy S., Bahal R., Ly D.H. (2011). Strand invasion of mixed-sequence, double-helical B-DNA by gamma-peptide nucleic acids containing g-clamp nucleobases under physiological conditions. Biochemistry.

[16] Bahal R., Sahu B., Rapireddy S., Lee C.M., Ly D.H. (2012). Sequence-unrestricted, Watson-Crick recognition of double helical B-DNA by (*R*)-MiniPEG-γPNAs. Chem. Bio. Chem..

[17] Gaj T., Gersbach C.A., Barbas C.F. (2013). ZFN, TALEN, and CRISPR/Cas-based methods for genome engineering. Trends Biotechnol..

[18] Didion B.A., Karmakar S., Guenther D.C., Sau S.P., Verstegen J.P., Hrdlicka P.J. (2013). Invaders: Recognition of double-stranded DNA using duplexes modified with interstrand zippers of 2′-*O*-(pyren-1-yl)methylribonucleotides. Chem. Biol. Chem..

[19] Guenther D.C., Anderson G.H., Karmakar S., Anderson B.A., Didion B.A., Guo W., Verstegen J.P., Hrdlicka P.J. (2015). Invader probes: Harnessing the energy of intercalation to facilitate recognition of chromosomal DNA for diagnostic applications. Chem. Sci..

[20] Sau S.P., Madsen A.S., Podbevsek P., Andersen N.K., Kumar T.S., Andersen S., Rathje R.L., Anderson B.A., Guenther D.C., Karmakar S. (2013). Identification and characterization of 2nd generation Invader LNAs for mixed-sequence recognition of double-stranded DNA. J. Org. Chem..

[21] Karmakar S., Madsen A.S., Guenther D.C., Gibbons B.C., Hrdlicka P.J. (2014). Recognition of double-stranded DNA using energetically activated duplexes with interstrand zippers of 1-, 2- or 4-pyrenyl-functionalized *O*2ʹ-alkylated RNA monomers. Org. Biomol. Chem..

[22] Crothers D.M. (1968). Calculation of binding isotherms for heterogeneous polymers. Biopolymers.

[23] Persil O., Hud N.V. (2007). Harnessing DNA intercalation. Trends Biotechnol..

[24] Tsai C., Jain S.C., Sobell H.M. (1977). Visualization of drug-nucleic acid interactions at atomic resolution. Structure of an ethidium-dinucleoside monophosphate crystalline complex ethidium—5-iodouridylyl (3′-5′) adenosine. J. Mol. Biol..

[25] Williams L.D., Egli M., Gao Q., Rich A., Sarma R.H., Sarma M.H. (1992). Structure and Function, Volume 1: Nucleic Acids.

[26] Sau S.P., Kumar T.S., Hrdlicka P.J. (2010). Invader LNA—Efficient targeting of short DNA duplexes. Org. Biomol. Chem..

[27] Anderson B.A., Onley J.J., Hrdlicka P.J. (2015). Recognition of double-stranded DNA using energetically activated duplexes modified with *N*2′-pyrene-, perylene-, or coronene-functionalized 2′-*N*-methyl-2′-amino-DNA monomers. J. Org. Chem..

[28] Karmakar S., Guenther D.C., Hrdlicka P.J. (2013). Recognition of mixed-sequence DNA duplexes: Design guidelines for Invaders based on 2′-*O*-(pyren-1-yl)methyl-RNA monomers. J. Org. Chem..

[29] Campbell J.M., Bacon T.A., Wickstrom E. (1990). Oligodeoxynucleoside phosphotothioate stability in subcellular extracts, culture media, sera and cerebrospinal fluid. J. Biochem. Biophys. Methods.

[30] Eckstein F. (2014). Phosphorothioates, essential components of therapeutic oligonucleotides. Nucleic Acid Ther..

[31] Stein C.A., Subasinghe C., Shinozuka K., Cohen J.S. (1988). Physicochemical properties of phosphorothioate oligodeoxynucleotides. Nucleic Acids Res..

[32] Karmakar S., Anderson B.A., Rathje R.L., Andersen S., Jensen T., Nielsen P., Hrdlicka P.J. (2011). High-affinity DNA-targeting using readily accessible mimics of *N*2′-functionalized 2′-amino-α-l-LNA. J. Org. Chem..

[33] Nakamura M., Fukunaga Y., Sasa K., Ohtoshi Y., Kanaori K., Hayashi H., Nakano H., Yamana K. (2005). Pyrene is highly emissive when attached to the RNA duplex but not to the DNA duplex: The structural basis of this difference. Nucleic Acids Res..

[B34-molecules-20-13780] 34.For a discussion of binding specificities of **X**-/**Y**-modified PS-DNA, see the Supporting Information (Tables S3 and S4).

[35] Asanuma H., Fujii T., Kato T., Kashida H. (2012). Coherent interactions of dyes assembled on DNA. J. Photochem. Photobiol. C.

[36] Dougherty G., Pilbrow J.R. (1984). Physicochemical probes of intercalation. Int. J. Biochem..

[37] Manoharan M., Tivel K.L., Zhao M., Nafisi K., Netzel T.L. (1995). Base-sequence dependence of emission lifetimes for dna oligomers and duplexes covalently labeled with pyrene—Relative electron-transfer quenching efficiencies of A-nucleoside, G-nucleoside, C-nucleoside, and T-nucleoside toward pyrene. J. Phys. Chem..

[38] Wilson J.N., Cho Y., Tan S., Cuppoletti A., Kool E.T. (2008). Quenching of fluorescent nucleobases by neighboring DNA: The “Insulator” concept. Chem. Biol. Chem..

[B39-molecules-20-13780] 39.For data on DNAse I stability, see Figure S7 in the Supporting Information.

[40] Dioubankova N.N., Malakhov A.D., Stetsenko D.A., Gait M.J., Volynsky P.E., Efremov R.G., Korshun V.A. (2003). Pyrenemethyl ara-uridine-2ʹ-carbamate: A strong interstrand excimer in the major groove of a DNA duplex. Chem. Biol. Chem..

[41] Brown A.M. (2001). A step-by-step guide to non-linear regression analysis of experimental data using a Microsoft Excel spreadsheet. Comput. Meth. Prog. Biomed..

