# Supplementary material for: Mixed-Sequence Recognition of Double-Stranded DNA Using Enzymatically Stable Phosphorothioate Invader Probes
Source: Molecules. 2015 Jul 29;20(8):13780–93. doi: 10.3390/molecules200813780 (PMC6332310; doi:10.3390/molecules200813780)
Supplement: Supplementary file 1 [file molecules-20-13780-s001.pdf]

Supporting Information

MALDI-MS of modified ONs (Table S1) S2

Representative thermal denaturation profiles (Figure S1) S3

DNA selectivity of **X**- and **Y**-modified 9-mer ONs (Table S2) S3

Discrimination of mismatched DNA targets by **X2** and **Y2** (Table S3) S4

Discrimination of mismatched DNA targets by **X6** and **Y6** (Table S4) S4

Absorption spectra of **X1**–**X6** in absence or presence of cDNA or cRNA (Figure S2) S5

Absorption spectra of **Y1**–**Y6** in absence or presence of cDNA or cRNA (Figure S3) S6

Absorbance of **X-/Y-**modified PS-DNA in absence/presence of cDNA/cRNA (Table S5) S7

Steady-state fluorescence emission spectra of **X5** and **Y5** in absence or presence of cDNA or cRNA (Figure S4) S7

Thermal advantage (*TA*) values for 13-mer PS-DNA/PO-DNA Invader probes (Table S6) S8

Recognition of dsDNA model target **DH1** using **X2**:**X5** or **Y2**:**Y5** (Figure S5) S9

Recognition of dsDNA model target **DH2** using different 13-mer Invader probes (Figure S6) S10

DNase I protocol and discussion S11

Nuclease susceptibility of PO-Invader probes in the presence of DNase I (Figure S7) S11

Supplementary references S12

Table S1. MALDI-MS of modified PS-DNA [a].

ON	PS-DNA Sequence	Calc. *m*/*z* [M + H]$^{+}$	Found *m*/*z* [M + H]$^{+}$
**X1**	5′-G**X**G ATA TGC	3110.4	3110.3
**X2**	5′-GTG A**X**A TGC	3110.4	3110.4
**X3**	5′-GTG ATA **X**GC	3110.4	3110.2
**X4**	3′-CAC **X**AT ACG	3039.4	3039.2
**X5**	3′-CAC TA**X** ACG	3039.4	3039.5
**X6**	3′-CAC **X**A**X** ACG	3211.5	3212.0
**Y1**	5′-G**Y**G ATA TGC	3097.5	3097.2
**Y2**	5′-GTG A**Y**A TGC	3097.5	3097.4
**Y3**	5′-GTG ATA **Y**GC	3097.5	3097.2
**Y4**	3′-CAC **Y**AT ACG	3026.5	3026.3
**Y5**	3′-CAC TA**Y** ACG	3026.5	3026.4
**Y6**	3′-CAC **Y**A**Y** ACG	3242.5	3242.3
**X7**	5′-GG**X** ATA TAT AGG C	4433.5	4433.4
**X8**	3′-CCA **X**AT ATA TCC G	4313.5	4313.4
**X9**	5′-GG**X** A**X**A TAT AGG C	4662.6	4662.4
**X10**	3′-CCA **X**A**X** ATA TCC G	4542.6	4542.4
**X11**	5′- GGT A**X**A **X**AT AGG C	4662.6	4662.6
**X12**	3′-CCA TA**X** A**X**A TCC G	4542.6	4542.4
**X13**	5′-GG**X** ATA TA**X** AGG C	4662.6	4662.4
**X14**	3′-CCA **X**AT ATA **X**CC G	4542.6	4542.5
**Y7**	5′-GG**Y** ATA TAT AGG C	4420.5	4420.5
**Y8**	3′-CCA **Y**AT ATA TCC G	4300.5	4300.5
**Y9**	5′-GG**Y** A**Y**A TAT AGG C	4636.6	4636.6
**Y10**	3′-CCA **Y**A**Y** ATA TCC G	4516.5	4516.6
**Y11**	5′- GGT A**Y**A **Y**AT AGG C	4636.6	4636.6
**Y12**	3′-CCA TA**Y** A**Y**A TCC G	4516.5	4516.5
**Y13**	5′-GG**Y** ATA TA**Y** AGG C	4636.6	4636.8
**Y14**	3′-CCA **Y**AT ATA **Y**CC G	4516.5	4516.7

[a] For structure of monomers **X** and **Y** see Figure 1 in the main manuscript.

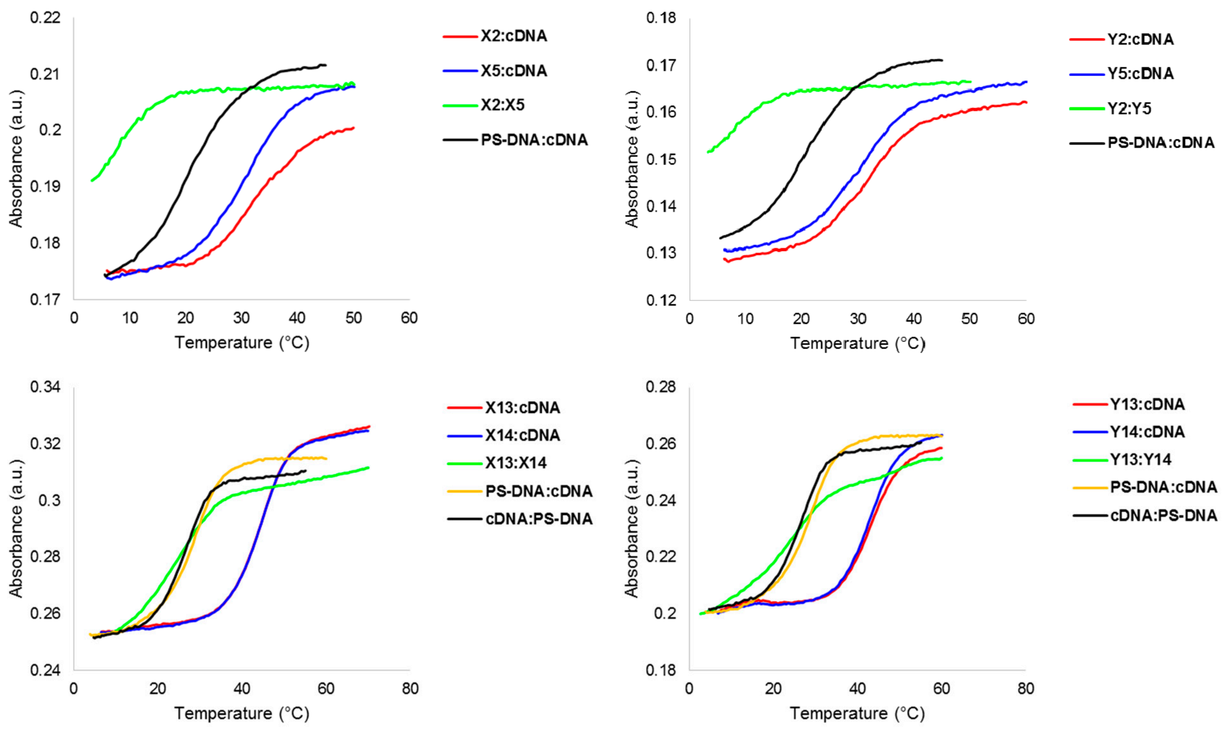

Figure S1. Representative thermal denaturation profiles of duplexes between **X**-/**Y**-modified PS-DNA strands and cDNA. For experimental conditions, see Table 1.

Table S2. DNA selectivity of **X**- and **Y**-modified PS-DNA [a].

ON	PS-DNA Sequence	$\Delta\Delta T_m$ (DNA-RNA) (°C)
**X1**	5′-G**X**G ATA TGC	+9.0
**X2**	5′-GTG A**X**A TGC	+13.0
**X3**	5′-GTG ATA **X**GC	+12.0
**X4**	3′-CAC **X**AT ACG	+9.0
**X5**	3′-CAC TA**X** ACG	+9.5
**X6**	3′-CAC **X**A**X** ACG	>+19.0
**Y1**	5′-G**Y**G ATA TGC	+8.5
**Y2**	5′-GTG A**Y**A TGC	+12.0
**Y3**	5′-GTG ATA **Y**GC	+9.5
**Y4**	3′-CAC **Y**AT ACG	+7.5
**Y5**	3′-CAC TA**Y** ACG	+9.5
**Y6**	3′-CAC **Y**A**Y** ACG	+17.0

[a] DNA selectivity defined as $\Delta\Delta T_m$ (DNA-RNA) = ΔT_m(*vs.* cDNA) − ΔT_m(*vs.* cRNA).

Binding Specificity of X-/Y-modified PS-DNA

The binding specificities of centrally modified 9-mer PS-DNA strands were studied using DNA targets with mismatched nucleotides opposite to the modification (Table S3). Excellent discrimination of the C-mismatched target is observed, while discrimination of G- or T-mismatched targets is much less efficient. On the other hand, doubly modified 9-mer PS-DNA discriminate DNA targets with a single mismatched nucleotide opposite of the central 2′-deoxyriboadenosine very efficiently (Table S4). These trends mirror our observations with **X**-/**Y**-modified PO-DNA strands, further suggesting that the pyrene moieties are intercalating upon cDNA hybridization [1,2].

Table S3. Discrimination of mismatched DNA targets by **X2** and **Y2** [a].

ON	PS-DNA Sequence	B =	DNA: 3′-CAC TBT ACG			
			T_m (°C)	ΔT_m (°C)		
			A	C	G	T
**X2**	5′-GTG A**X**A TGC		**32.5**	−16.0	−2.5	−9.0
**Y2**	5′-GTG A**Y**A TGC		**31.5**	−13.0	−5.5	−7.5

[a] For conditions of thermal denaturation experiments, see Table 1. T_m's of fully matched duplexes are shown in bold. ΔT_m = change in T_m relative to fully matched DNA:DNA duplex.

Table S4. Discrimination of mismatched DNA targets by **X6** and **Y6** [a].

ON	PS-DNA Sequence	B =	DNA: 5′-GTG ABA TGC			
			T_m (°C)	ΔT_m (°C)		
			T	A	C	G
**X6**	3′-CAC **X**A**X** ACG		**30.5**	<−20.5	−13.0	−14.0
**Y6**	3′-CAC **Y**A**Y** ACG		**30.0**	−18.5	−16.0	−13.5

[a] For conditions of thermal denaturation experiments, see Table 1. T_m's of fully matched duplexes are shown in bold. ΔT_m = change in T_m relative to fully matched DNA:DNA duplex.

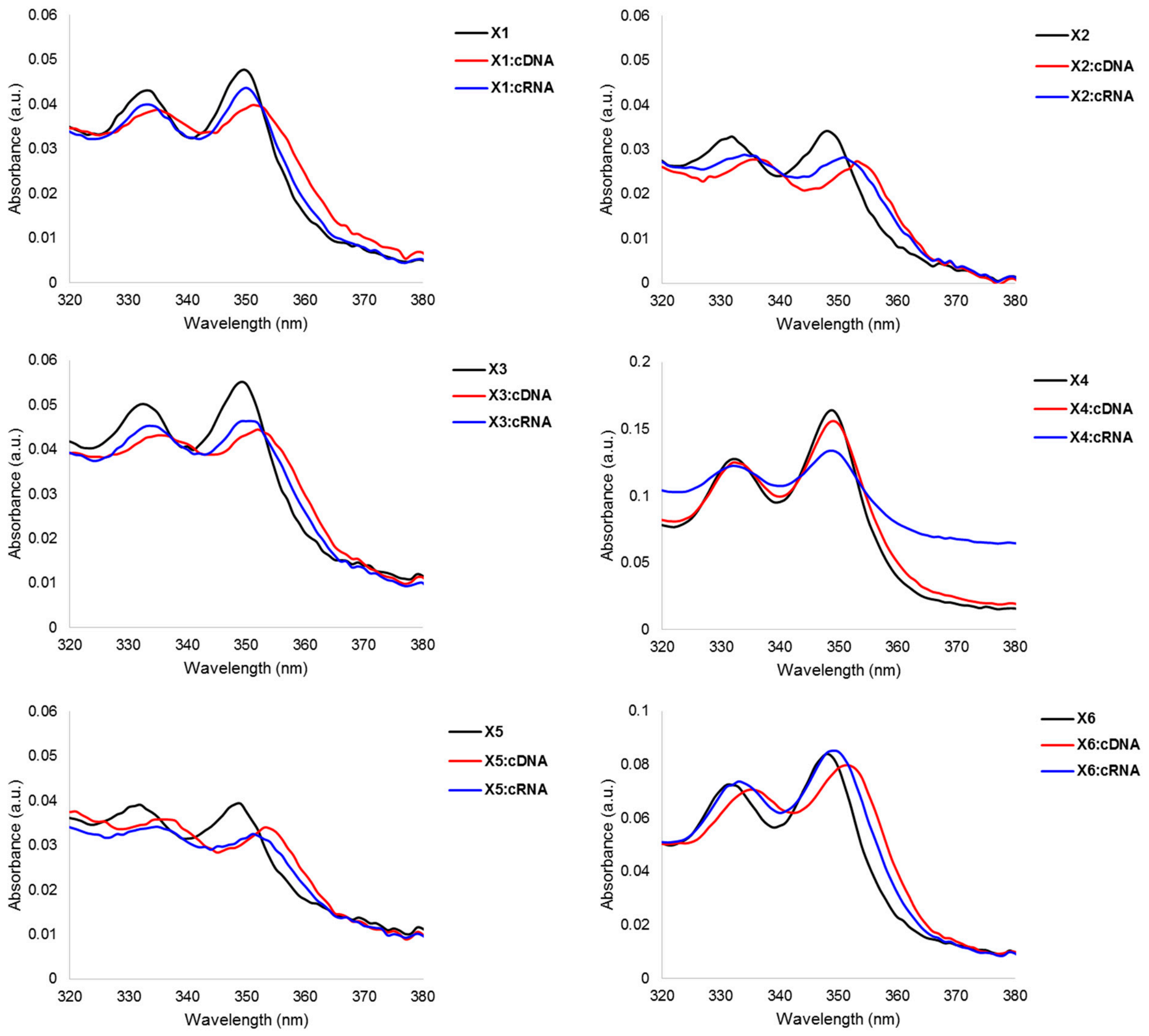

Figure S2. Absorption spectra of **X1**–**X6** in absence or presence of cDNA/cRNA. Spectra were recorded at T = 5 °C using each strand at 1.0 µM concentration in T_m buffer. Note, different axis scales are used.

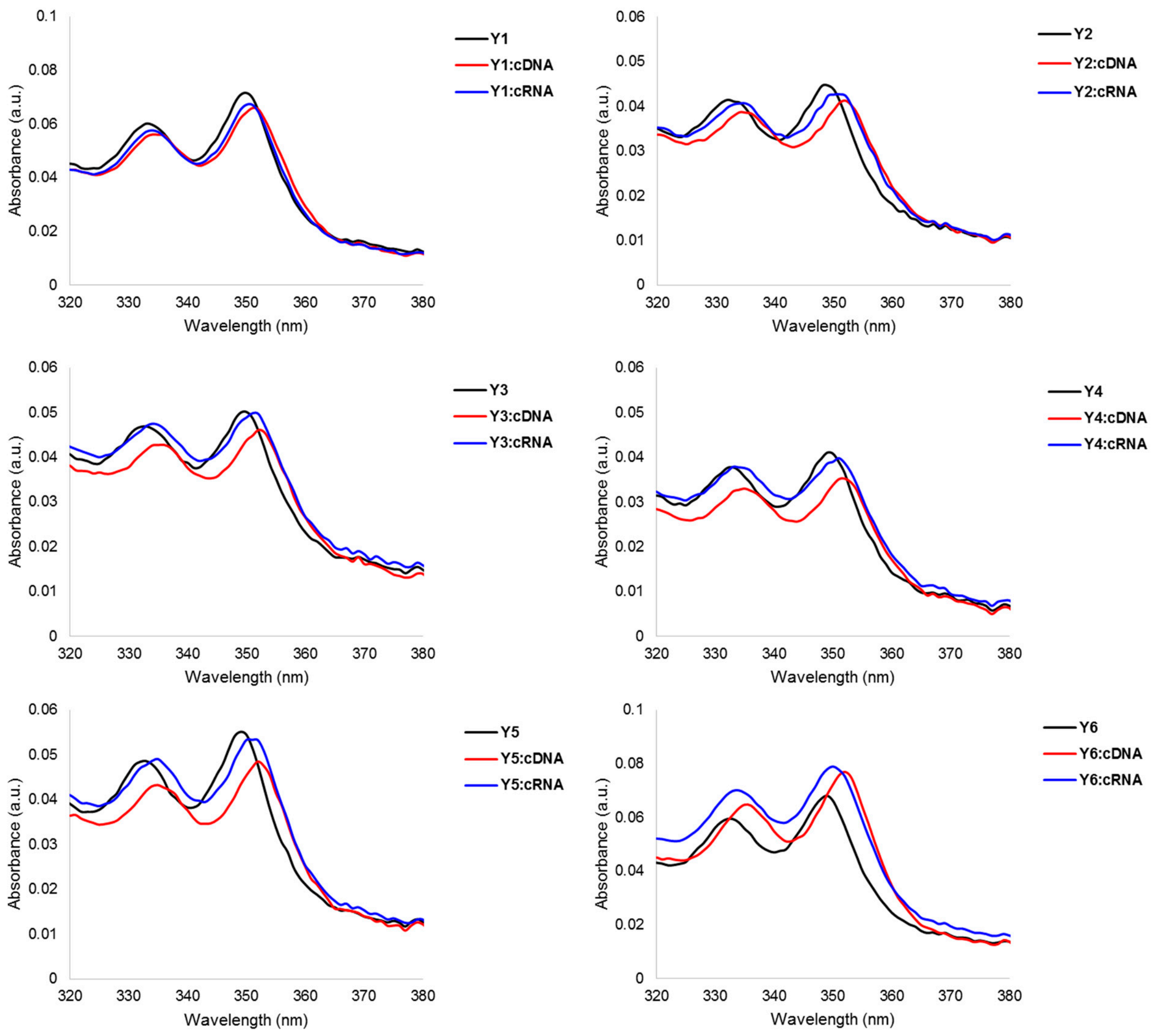

Figure S3. Absorption spectra of **Y1**–**Y6** in absence or presence of cDNA/cRNA. Spectra were recorded at T = 5 °C using each strand at 1.0 µM concentration in T_m buffer. Note, different axis scales are used.

Table S5. Absorption maxima in the 300–500 nm region for **X**- and **Y**-modified PS-DNA and the corresponding duplexes with cDNA or cRNA [a].

ON	PS-DNA Sequence	λ_{max} [$\Delta\lambda_{max}$]/nm		
		SSP	+cDNA	+cRNA
**X1**	5′-G<u>**X**</u>G ATA TGC	350	351 [+1]	350 [±0]
**X2**	5′-GTG A<u>**X**</u>A TGC	348	353 [+5]	351 [+3]
**X3**	5′-GTG ATA <u>**X**</u>GC	349	352 [+3]	351 [+2]
**X4**	3′-CAC <u>**X**</u>AT ACG	349	349 [±0]	349 [±0]
**X5**	3′-CAC TA<u>**X**</u> ACG	349	353 [+4]	351 [+2]
**X6**	3′-CAC <u>**X**</u>A<u>**X**</u> ACG	348	351 [+3]	349 [+1]
**Y1**	5′-G<u>**Y**</u>G ATA TGC	350	351 [+1]	351 [+1]
**Y2**	5′-GTG A<u>**Y**</u>A TGC	349	352 [+3]	351 [+2]
**Y3**	5′-GTG ATA <u>**Y**</u>GC	350	352 [+2]	351 [+1]
**Y4**	3′-CAC <u>**Y**</u>AT ACG	349	352 [+3]	351 [+2]
**Y5**	3′-CAC TA<u>**Y**</u> ACG	349	352 [+3]	351 [+2]
**Y6**	3′-CAC <u>**Y**</u>A<u>**Y**</u> ACG	349	352 [+3]	350 [+1]

[a] SSP = single-stranded probe. Measurements were performed at 5 °C using a spectrophotometer and quartz optical cells with 1.0 cm path lengths. For buffer conditions, see Table 1.

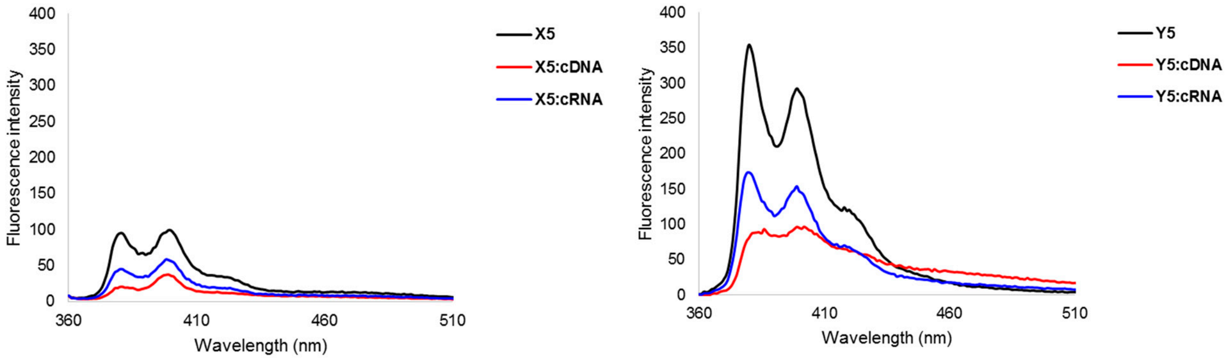

Figure S4. Representative fluorescence spectra of single-stranded **X5** and **Y5** and the corresponding duplexes with cDNA/cRNA. Spectra were recorded at T = 10 °C using λ_{ex} = 350 nm. Each strand was used at 1.0 μM concentration in T_m buffer.

Table S6. Thermal advantage (*TA*) values for 13-mer PS-DNA/PO-DNA Invader probes [a].

ON	Invader Probe	**B** =	PS-DNA Backbone		PO-DNA Backbone	
			TA/°C		*TA*/°C	
			**X**	**Y**	**X** [b]	**Y** [b]
**B7**	5′-GG**B** ATA TAT AGG C		20	20	22	18
**B8**	3′-CCA **B**AT ATA TCC G					
**B9**	5′-GG**B** A**B**A TAT AGG C		28.5	23	25	29.5
**B10**	3′-CCA **B**A **B**ATA TCC G					
**B11**	5′- GGT A**B**A **B**AT AGG C		34.5	25.5	35	28.5
**B12**	3′- CCA TA**B** A**B**A TCC G					
**B13**	5′-GG**B** ATA TA**B** AGG C		27	23.5	32	21.5
**B14**	3′-CCA **B**AT ATA **B**CC G					

[a] The term *thermal advantage* (*TA*) = T_m (5′-Inv:cDNA) + T_m (3′-Inv:cDNA) − T_m (Invader probe) − T_m (dsDNA target)], serves as a first approximation to describe the energy difference between the 'products' and 'reactants' of the prototypical recognition process, with more positive values signifying greater thermodynamic dsDNA recognition potential [3]. See Table 1 in main manuscript for T_m's of 5′-Inv:cDNA and 3′-Inv:cDNA. See Table 2 in the main manuscript for T_m's of Invader probes. T_m of the isosequential dsDNA target is 37.5 °C [3]. [b] Data from reference [3].

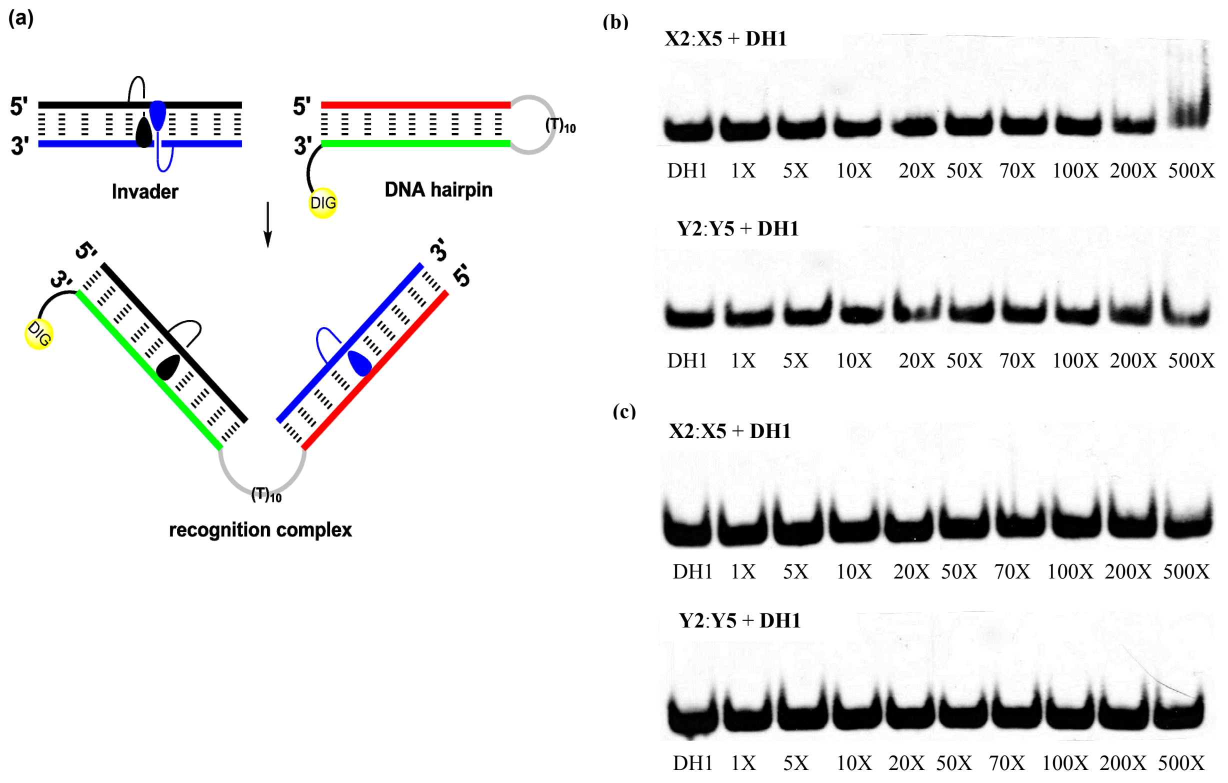

Figure S5. Recognition of dsDNA model target **DH1** using **X2**:**X5** or **Y2**:**Y5**. (**a**) Illustration of recognition process. Sequence of **DH1**: 5′-GTGATATGC-(T_{10})-GCTTATCAC-DIG-3′; (**b**) representative gel electrophoretograms from experiments in which **DH1** (34.4 nM) was incubated with **X2**:**X5** or **Y2**:**Y5** at ambient temperature for 12-16 h; (**c**) representative gel electrophoretograms from experiments in which **DH1** (34.4 nM) was annealed in the presence of **X2**:**X5** or **Y2**:**Y5** at 85 °C for 15 min, followed by cooling to room temperature over ~30 min and incubation at ambient temperature for 12–16 h. Experiments were conducted in 1X HEPES buffer (50 mM HEPES, 100 mM NaCl, 5 mM $MgCl_2$, 10% sucrose, 1.4 mM spermine tetrahydrochloride, pH 7.2) and then run on 16% non-denaturing PAGE (performed at 70 V, 2.5 h, ~4 °C) using 0.5× TBE as a running buffer (45 mM Tris, 45 mM boric acid, 1 mM EDTA); DIG: digoxigenin.

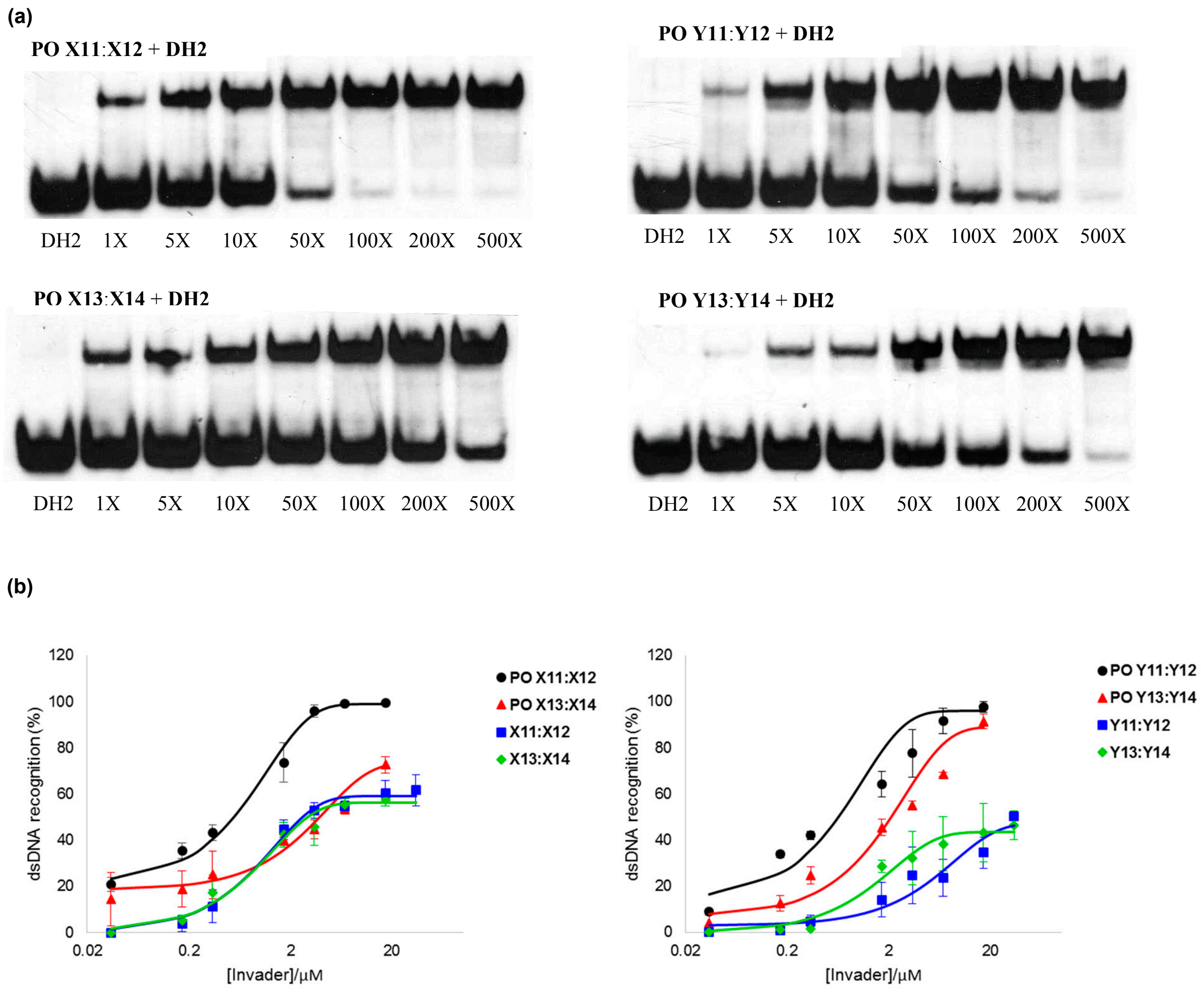

Figure S6. Recognition of dsDNA model target **DH2** using different Invader probes. (**a**) Representative electrophoretograms for recognition of **DH2** using 1- to 500-fold excess of the PO-DNA versions of **X11**:**X12**, **X13**:**X14**, **Y11**:**Y12**, **Y13**:**Y14**; (**b**) dose-response curves (average of at least three independent experiments, error bars represent standard deviation) relative to the corresponding PS-DNA Invaders. The sequence of DNA hairpin **DH2** and experimental are given in Figure 3.

Protocol—Stability of Invader Probes against DNase 1

An aqueous solution of DNase I (Worthington Biochemical Corporation—0.61 µL of a 2 µg/mL solution) was added to a 6.3 µM solution of a specific pre-annealed Invader in TE buffer (100 µL, 10 mM Tris·HCl, 0.1 mM EDTA, 10 mM $MgCl_2$, pH 8.0) and the mixture was incubated at 20 °C in a water bath. Aliquots (10 µL) were removed at specific times (0.5, 1, 2, 5, 10, 20 and 30 min) and degradation was quenched by addition of ethidium bromide buffer (2.0 mL, 5 mM Tris·HCl, 0.5 mM EDTA, 0.5 µg/mL EtBr, pH 8.0). The fluorescence intensity of the solution was measured (λ_{ex} = 525 nm; λ_{em} = 600 nm) using the same instrumentation employed for the steady-state fluorescence experiments. Intensities were averaged over 15 minutes. Experiments were performed in duplicates and representative graphs are shown.

Discussion—Stability of Invader Probes against DNase 1

The stability of Invader probes against DNase I was evaluated using an ethidium bromide based assay. In this assay, high levels of fluorescence are observed when the studied duplex is intact due to intercalating ethidium bromide, while low levels of fluorescence are expected if a duplex has been degraded to single strands (or shortened to a level where the duplex dissociates) [4,5].

Similar assays have been used to show that long PS-DNA duplexes exhibit excellent stability against DNase I [6,7]. Figure S7 shows the fluorescence intensity profiles of representative 13-mer Invaders in the presence of DNase I. The PO-DNA analogs of **X13**:**X14** and **Y13**:**Y14** are moderately resistant to DNase I degradation with half-lives of 15 min and >30 min, respectively, whereas the unmodified PO-DNA duplex is rapidly degraded.

PS-DNA Invaders **X13**:**X14** and **Y13**:**Y14** did not show any change in fluorescence emission relative to background EtBr buffer (data not shown), presumably because the probe duplexes are dissociated into single strands at the experimental conditions used for this assay (T_m of **X13**:**X14** and **Y13**:**Y14** are <25 °C, Table 2).

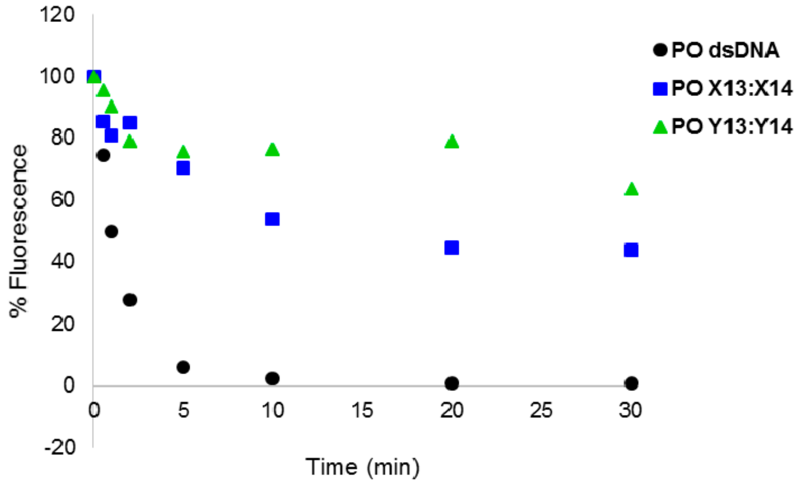

Figure S7. DNase I stability of PO-DNA Invader probes **X13**:**X14**, **Y13**:**Y14** and the corresponding unmodified PO-DNA duplex, as assessed by ethidium bromide assay. Curves are average of two experiments.

Supplementary References

1. Anderson, B.A.; Onley, J.J.; Hrdlicka, P.J. Recognition of double-stranded DNA using energetically activated duplexes modified with N2′-pyrene-, perylene-, or coronene-functionalized 2′-*N*-methyl-2′-amino-DNA monomers. *J. Org. Chem.* **2015**, *80*, 5395–5406.
2. Karmakar, S.; Anderson, B.A.; Rathje, R.L.; Andersen, S.; Jensen, T.; Nielsen, P.; Hrdlicka, P.J. High-affinity DNA-targeting using readily accessible mimics of N2′-functionalized 2′-amino-α-L-LNA. *J. Org. Chem.* **2011**, *76*, 7119–7131.
3. Guenther, D.C.; Anderson, G.H.; Karmakar, S.; Anderson, B.A.; Didion, B.A.; Guo, W.; Verstegen, J.P.; Hrdlicka, P.J. Invader probes: Harnessing the energy of intercalation to facilitate recognition of chromosomal DNA for diagnostic applications. *Chem. Sci.* **2015**, doi:10.1039/C5SC01238D.
4. Morgan, A.R.; Lee, J.S.; Pulleyback, D.E.; Murray, N.L.; Evans, D.H. Review: Ethidium fluorescence assays. Part 1. Physicochemical studies. *Nucleic Acids Res.* **1979**, *7*, 547–570.
5. Morgan, A.R.; Evans, D.H.; Lee, J.S.; Pulleyback, D.E. Review: Ethidium fluorescence assay. Part H. Enzymatic studies and DNA-protein interactions. *Nucleic Acids Res.* **1979**, *7*, 571–594.
6. Braun, R.P.; Lee, J.S. Immunogenic duplex nucleic acids are nuclease resistant. *J. Immun.* **1988**, *141*, 2084–2089.
7. Latimer, L.J.P.; Hampel, K.; Lee, J.S. Synthetic repeating sequence DNAs containing phosphorothioates: Nuclease sensitivity and triplex formation. *Nucleic Acids Res.* **1989**, *17*, 1549–1561.
